# Moho topography, ranges and folds of Tibet by analysis of global gravity models and GOCE data

**DOI:** 10.1038/srep11681

**Published:** 2015-06-26

**Authors:** Young Hong Shin, C.K. Shum, Carla Braitenberg, Sang Mook Lee, Sung -Ho Na, Kwang Sun Choi, Houtse Hsu, Young-Sue Park, Mutaek Lim

**Affiliations:** 1 Korea Institute of Geosciences and Mineral Resource, Daejeon, 305-350, Korea; 2 Division of Geodetic Science, School of Earth Sciences, Ohio State University, Columbus, Ohio 43210, USA; 3 Dept. of Mathematics and Geosciences, University of Trieste, Via Weiss 1, 34100 Trieste, Italy; 4 School of Earth & Environmental Sciences, Seoul National University, 151-742, Korea; 5 University of Science and Technology, Daejeon, 305-350, Korea; 6 Dept. of Earth Science, Pusan National University, 609-735, Korea; 7 Institute of Geodesy and Geophysics, Chinese Academy of Sciences, 130077 Wuhan, China

## Abstract

The determination of the crustal structure is essential in geophysics, as it gives insight into the geohistory, tectonic environment, geohazard mitigation, etc. Here we present the latest advance on three-dimensional modeling representing the Tibetan Mohorovičić discontinuity (topography and ranges) and its deformation (fold), revealed by analyzing gravity data from GOCE mission. Our study shows noticeable advances in estimated Tibetan Moho model which is superior to the results using the earlier gravity models prior to GOCE. The higher quality gravity field of GOCE is reflected in the Moho solution: we find that the Moho is deeper than 65 km, which is twice the normal continental crust beneath most of the Qinghai-Tibetan plateau, while the deepest Moho, up to 82 km, is located in western Tibet. The amplitude of the Moho fold is estimated to be ranging from −9 km to 9 km with a standard deviation of ~2 km. The improved GOCE gravity derived Moho signals reveal a clear directionality of the Moho ranges and Moho fold structure, orthogonal to deformation rates observed by GPS. This geophysical feature, clearly more evident than the ones estimated using earlier gravity models, reveals that it is the result of the large compressional tectonic process.

The Qinghai-Tibetan Plateau offers significant interest in geoscience because of its particularly high topography, extraordinary thick crust, its late Cenozic geologic uplift evolution, and interactions with regional climate change^1^,^2^,^3^,^4^. The most plausible explanation of the geophysical process is the continental collision between the Eurasian and Indian plates, although the details of the collisional process and related deformation still remain controversial. In concert with growth of the topography in the compressional environment, the crust thickens due to the isostatic compensation process. The lithosphere (including the crust and upper mantle) also undergoes buckling when the horizontal compression exceeds a critical value. Lithospheric folding is an important mode of basin formation in compressional intraplate settings^5^, and it may affect the generation of basaltic magma beneath the deforming lithosphere under some geophysical conditions^6^,^7^. Recent satellite-based gravimetric studies^1^,^2^ have revealed the three-dimensional (3D) structural features – the Moho ranges and fold, which agree well with expected directionality and amplitude of the deformed crustal structure in the compressional tectonic environment, and surface velocities measured by Global Positioning System (GPS) satellites^3^.

Although a number of international collaborative seismic and magnetotelluric explorations^8^,^9^,^10^,^11^,^12^,^13^,^14^ have been successful in revealing the subsurface structure, as disclosing the subducting slab, determining crust-mantle boundary, crustal rheology, etc., they are limited along a few cross-sectional lines. Like other geophysical methods, gravimetric inversion is limited by poor gravity data quality and sparse spatial coverage, due primarily to the precipitous and remote unapproachable topography. In particular, little or very few *in situ* gravity data are available to the scientific community over the Tibetan Plateau. The limitation has recently been partially overcome due to the advances in satellite gravity modeling using data from the Gravity Recovery and Climate Experiment (GRACE) mission, providing mean and temporal gravity field with unprecedented accuracy and spatial resolution at global scale. The contribution of GRACE, although at long wavelength (spherical harmonic degree 120 or lower), is obvious especially in the area of little, no or bad terrestrial gravity data. The Tibetan Plateau is one of these cases where the GRACE gravity field model contributed significantly to Moho fold studies^1^,^2^.

The most recent gravity mission satellite, the Gravity field and steady-state Ocean Circulation Explorer (GOCE), was launched by European Space Agency in March 2009 and finally ran out of fuel November 2013. It successfully recovered much shorter wavelength gravity signals than GRACE, as it was equipped with the state-of-the-art gravity gradiometer system and had flown at the lowest altitude that gravity mission satellites ever did, thereby maximizing its sensitivity to Earth’s gravity field. The satellite was operated at the desired altitude of 254 km for science operations from September 2009 to July 2012, while the altitude was lowered to the final value of 224 km in several steps from August 2012 to May 2013. The GOCE satellite-only global gravity field model is now complete to spherical harmonic degree 300, representing a spatial resolution of 0.6 arc-degrees in spherical coordinates. The most recent model, GO_CONS_GCF_2_DIR_R5 (released on 18 July 2014, hereafter DIR_R5) is used as the primary gravity model for this study and the other gravity models shown in Table S1 are used for comparative and accuracy assessment studies. In earlier studies, the GGM02C model^15^ was used for revealing the Tibetan Moho structure^2^, while the EIGEN-GL04C^16^ was used to study the Tibetan Moho folds^1^. Unlike the satellite-only model, DIR_R5, both the GGM02C and EIGEN-GL04C models are combination models using GRACE satellite observations and terrestrial gravity data. Therefore even though the maximum degree of the EIGEN-GL04C is 360, higher than any other satellite-only models, the gravity model in and around the Tibetan Plateau and its vicinity is dominated by satellite data at much lower resolution (degree 120)^1^,^2^,^17^. As a result, the advantage of the DIR_R5 and other satellite-only models is that the models are not contaminated by data outages in the Tibetan Plateau. The advantage is even clearer as the spatial resolution increases with the addition of the GOCE data.

The free-air gravity anomaly, at the reference height of 7-km above mean sea level, varies from –200 mGal to 232 mGal, with a mean of –7 mGal and a standard deviation of 58 mGal (Fig. S1). The edge effect, representing the combined effect of topography and deeply seated crust-mantle interface, is clearly found along the boundary of the plateau while the other areas have a rather small lateral gravity variation. The Bouguer anomaly, reflecting the subsurface density structure, varies from –559 mGal to 12 mGal, with a mean of –266 mGal and a standard deviation of 157 mGal (Fig. S1), through the reduction of the integrated gravity effect of topography and sediment. Significantly low gravity anomaly is observed inside the plateau, having east-west directionality, representing the deep crust-mantle boundary distributed beneath the region, while the surrounding areas have higher anomaly denoting thinner crust. The largest gravity differences with former gravity models are located along the southern and western border of the plateau (Fig. S2), coinciding with the area with absence of terrestrial gravity data^2^,^17^.

The Moho topography is computed using the gravity inversion with the DIR_R5 model and compared with earlier models (Fig. 1, Fig. S3). In our previous gravity inversion studies^18^,^19^, the density and reference depth were optimized such that the Moho results would best fit the constraining seismic lines, and we also adopt the parameters in the present study. The overall features of the Moho topography using the DIR_R5 model almost coincide with the previous two models^1^,^2^ using the EIGEN-GL04C and the GGM02C/EGM96. The Moho is generally deeper than 47 km beneath the Tibetan plateau, while the maximum depth, 82 km, is found in western Tibet and the minimum depth, 29 km, near the Dauki Thrust, which is beyond the study area. At the inner border of the plateau, the Moho is deeper than 65 km (depicted with red dashed line), twice of the normal continental crust. The Moho is found to be very deep in western and central Tibet with three east-west trending troughs, the so-called Moho ranges^2^, and becomes relatively shallower towards eastern Tibet. In addition, the Moho depth beneath the Tarim basin ranges from 34 km to 47 km, much shallower than the surrounding Tibetan plateau and the Tian Shan range. The Bachu uplift^20^,^21^, one of the major oil prospect areas in the Tarim basin, is clearly identified with the NW-SE oriented Moho shallowing to less than 38 km.

The improvement of the Moho topography model derived from the DIR_R5 gravity model is easily identified by the Moho ranges having better defined directionality than the previous two Moho models, using the EIGEN-GL04C and the GGM02C/EGM96 gravity models, respectively. The difference of the Moho topography models from the previous models (Fig. S3) shows a similar pattern as the gravity anomalies (Fig. S2) having large differences along the area of no-terrestrial gravity data, but the former do not have shorter wavelengths due to the smoothing process that excludes the upper crustal signals. Statistics of a good number of Moho topography models derived from the selected global gravity models are listed in Table S2 and their comparisons with the Moho derived using the DIR_R5 model in Table S3. The amplitudes of the overall Moho topography models are similar to that of the DIR_R5 model, while systematically smaller ones are found in the models developed during 2010, i.e., the ITG-GRACE2010S, GOCO01S, EIGEN-51C, and GO_CONS_GCF_2_DIR_R1 models. It might be caused by strong filtering to the GRACE data when the ITG-GRACE2010S model was derived, and the other models (GOCO01S, EIGEN-51C, and GO_CONS_GCF_2_DIR_R1) might also be affected by adopting this model for combination with GRACE data. However the features of the difference of Moho topography models (Table. S3) has quite different patterns than that of the Moho topography models (Table S2): it shows the chronological improvement of the models with respect to that of the DIR_R5, while the older model ITG-GRACE03S, released in 2007, has exceptionally better performance than latter models. It is noticeable that the most dramatic improvement appeared in the models since the availability of GOCE mission data in 2010, except the early GOCE models, GOCO01S and GO_CONS_GCF_2_DIR_R1.

The estimated Moho fold model from the most recent gravity model, the DIR_R5, is presented in Fig. 2, a comparison with the previous model from EIGEN-GL04C in Fig. S5. As the first Moho fold model beneath the Tibetan plateau^1^ had shown good coincidence with the present-day surface velocities measured by GPS^3^ and with the predicted wavelength of the fold formation from an elastic plate under horizontal compression, here the same method and parameters are applied to investigate the potential improvement of Moho fold models with the recent global gravity models. We find that the directionality of the Moho fold becomes more evident than before by reducing discontinuities due to isolated highs and lows of the former model^1^. The folds strike EW in the Central-Western Tibetan plateau, but rotate clock wise around the eastern Himalaya Syntaxis. The western Syntaxis is at the limit of our model, but also here the folds seem to rotate around the Syntaxis. The Moho ridges and troughs are orthogonal to the prevailing horizontal deformation rates recovered from GPS^3^,^22^. In eastern Tibet towards the China craton, the prevailing pattern turns from NS to NNE-SSW, when moving from central-east Tibet towards the Sichuan basin, following the main directions of horizontal velocity rates.

Taking the Moho fold model derived from DIR_R5 as a reference, Fig. 3 depicts the differences to the Moho models derived from other gravity models. Assuming that the most recent global gravity model, DIR_R5 has the best signals for the Tibetan Moho topography, it shows that the global gravity models have improved year after year and Moho fold models have converged, i.e. little changes since 2011 or 2012. It also seems that there was a jump of improvement with the availability of the GOCE mission data since 2010. The N-S directional stripe patterns, due to aliasing problem of GRACE data, are widely found in the models in which the GRACE data are included. This pattern is still present in the GRACE satellite-only models released in 2007 (ITG-GRACE03S), 2008 (GGM03S), and even in the most recent model in 2014 (GGM05S), while it is hardly found in the model in 2010 (ITG-GRACE2010S). The latter model could have been smoothed more than the other ones. In Fig. 3, Moho fold structure-like patterns are found in the models released in 2010, i.e. ITG-GRACE2010S, GOCO01S, EIGEN-51C, and GO_CONS_GCF_2_DIR_R1, which means that the Moho signals were lost in these models due to the too strong filter applied to their base model, ITG-GRACE2010S. In the earlier combination models before GOCE, the largest differences are observed along the no-terrestrial gravity data zone (i.e. west and south boundary of the Tibetan plateau), which means that the models are still suffering from the poor quality of surface gravity data. However it seems to have been greatly reduced since the introduction of the GOCE mission data.

Statistics of Moho fold models and their differences with the reference model, DIR_R5, are shown in Table S4 and S5, respectively. It seems that the numerical values of Moho fold models have converged: recent models show that the Moho fold oscillates between –9 to 9 km with respect to the long wavelength Moho, with a standard deviation of 2 km. A large difference is observed between the most recent Moho fold model of DIR_R5 and the previous model of EIGEN-GL04C^1^. It demonstrates a noticeable progress has been achieved for solid earth geophysics studies through the GOCE mission. The improvement of GOCE in the identification of geologic structures has been found also in other areas, as South America^23^,^24^ Africa^25^ and in the Alpine range^26^, next to the insights that could be achieved for the investigations of the mantle^27^.

We showed that both the Moho topography and fold model from the GOCE mission-based gravity model, DIR_R5, confirms the improvement of the gravity model by revealing the more evident directionality of folds compared with the previous models. Thus the 3D Tibetan Moho topography, ranges, and fold model of this study could be, at present, considered the state-of-the-art solution for the Tibetan crust, and could influence the study of other collisional boundaries.

## Methods

The global gravity field models we have applied are expressed in terms of spherical harmonics:


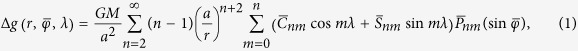


where *a* is the mean Earth equatorial radius, *GM* is the geocentric gravitational constant multiplied by the mass, 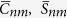 are the fully normalized spherical potential coefficients, 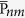 is the fully normalized Legendre function, and 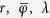 geocentric radius, latitude, longitude, respectively. For the purpose of suppressing the high-frequency error caused by shallow layer uncertainty of terrain and sediment, the reference level is set to 7-km height above mean sea level (MSL), a little higher than maximum topography, rather than the normally used MSL. Thus the upward continuation to a certain height, *h*, is simply computed by substituting the value *r* by *a* + *h* in the formula^28^.

The gravity effect on a point *P*(*r, θ, λ*) caused by a mass element *dM*(*r*′*, θ*′*, λ*′) in spherical coordinates is calculated with the formula: 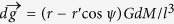, where *G* is the Newton’s gravitational constant, *dM* = *ρr*'^2^ sin*θ*'dr'd*θ*'d*λ*', *ρ* density, and *ψ* angular distance. Thus the Bouguer anomaly is computed by eliminating the gravity effect of the terrain above MSL and by filling up the mass deficiency in ocean and sedimentary basin^2^. We accepted the same method and parameters of the former studies^1^,^2^: e.g. integrating radius of 5 degree in angular distance and densities of 2,670 kg/m^3^, 1,030 kg/m^3^, and 200 kg/m^3^ for continent, ocean, and density contrast of sediments, respectively.

For the gravity inversion we apply the so-called Parker-Oldenburg method^29^,^30^ and a FORTRAN program^31^. The program is based on the modified relation of the Parker’s formula between the vertical gravity effect, Δ*g* and its causative mass topography, 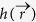 in Fourier domain^31^:


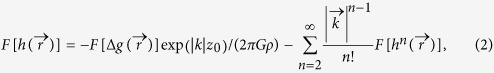


where 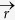 denotes the projection of the position r = (x, y, z) onto the x−y plane, 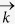 the wave vector of the transformed function, *G* the Newton’s gravitational constant, *ρ* density.

Finally, we determined the Moho fold by adopting the assumption that ‘the undulations of the Moho are mainly caused by vertical and horizontal loadings, which result in isostatic crustal thickening and buckling (folding), respectively. Therefore the deviation of the current Moho from the isostatic equilibrium can be largely explained by the fold structure in a collision environment, where the horizontal compression is the dominant force^1^. Although both the Airy-type isostasy and flexural response could be considered for the analysis of isostatic equilibrium, we prefer the latter, as the former assumes the unrealistic zero rigidity value of the lithosphere. Here we applied the coherence technique of Forsyth^32^ in analyzing the flexural isostasy. As a result, the folding patterns derived from the GOCE observations correlate very well to the GPS derived crustal velocities, with folding being preferentially orthogonal to the prevailing crustal velocity.

## Additional Information

**How to cite this article**: Shin, Y. H. *et al.* Moho topography, ranges and folds of Tibet by analysis of global gravity models and GOCE data. *Sci. Rep.* **5**, 11681; doi: 10.1038/srep11681 (2015).

## Supplementary Material

Supplementary Information

## Figures and Tables

**Figure 1:**
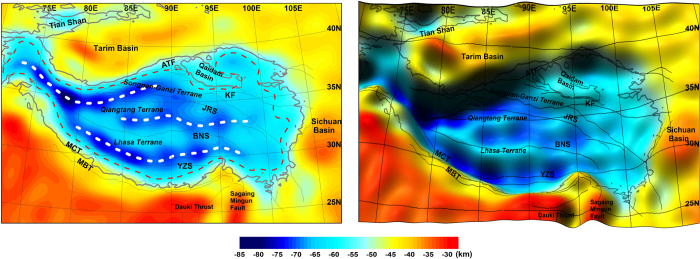
Moho topography from global gravity model, GO_CONS_GCF_2_DIR_R5 (left) and its shaded relief map (right), respectively: Grey line represents 3-km-height level to outline the plateau, while the red dashed line the 65 km depth Moho isoline. Moho ranges, depicted with white dashed lines, fit the deep trough belts of the model from GO_CONS_GCF_2_DIR_R5. Tectonic lines are labeled with acronyms as MBT (Main Himalaya Thrust), MCT (Main Central Thrust), YZS (Yarlung-Zangbo Suture), BNS (Bangong-Nujiang Suture), JRS (Jinsha River Suture), KF (Kunlun Fault), and ATF (Altyn Tagh Fault). (The figure is generated using Surfer (http://goldensoftware.com/) by Young Hong Shin).

**Figure 2:**
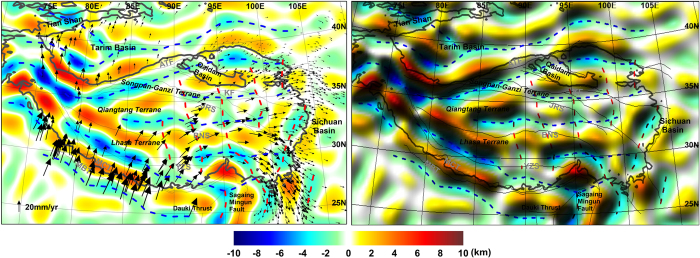
Moho fold models from GO_CONS_GCF_2_DIR_R5 (left) and its shaded relief maps (right), respectively: EW and NS directionality of Moho fold troughs are depicted with blue and red dashed lines, respectively. Blank arrows represent surface movement from GPS data^3^. (The figure is generated using Surfer (http://goldensoftware.com/) by Young Hong Shin).

**Figure 3:**
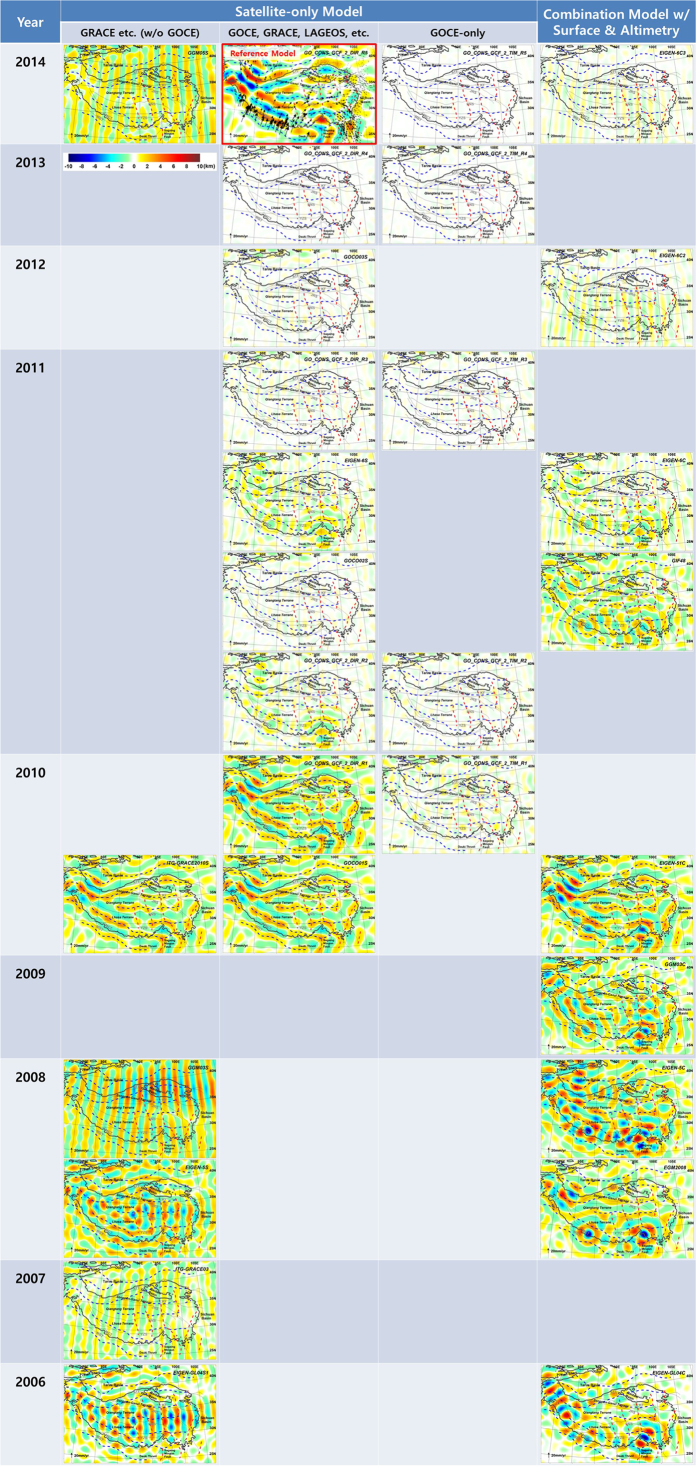
Difference of Moho fold models of various global gravity models with respect to that of GO_CONS_GCF_2_DIR_R5 (Reference Model). (The figure is generated using Surfer (http://goldensoftware.com/) by Young Hong Shin).
