# Supplementary material for: Moho topography, ranges and folds of Tibet by analysis of global gravity models and GOCE data
Source: Sci Rep. 2015 Jun 26;5:11681. doi: 10.1038/srep11681 (PMC4481824; doi:10.1038/srep11681)
Supplement: Supplementary Information [file srep11681-s1.doc]

**Supplementary Information for**

**Moho topography, ranges and folds of Tibet by analysis of global gravity models and GOCE data**

*by Young Hong Shin, C.K. Shum, Carla Braitenberg, Sang Mook Lee, Sung -Ho Na, Kwang Sun Choi, Houtse Hsu, Young-Sue Park, Mutaek Lim*

**1. Data and methodology**

Numerous global gravity field models, which have been developed since 1966, are collected and made available to the public by the International Centre for Global Earth Models (ICGEM, <http://icgem.gfz-potsdam.de/ICGEM/>). It was established as one of six centers of the International Gravity Field Service (IGFS) of the International Association of Geodesy (IAG) in 2003. Table S1 shows some of the available gravity field models since 2004. The models are classified into two groups: satellite-only models and combination models, while the satellite-only models are divided further into three subgroups: non-GOCE models, GOCE-combination models, and GOCE-only models. The time span of the satellite data and the maximum spherical harmonic degree of the models is listed according to the model name and release year.

The spatial resolution of the spherical harmonic models, expressed in terms of degree and order, is generally higher in combination models than satellite-only models, because the higher degree terms of the combination models, insensitive to satellites, were supplemented by land gravity data, airborne/shipboard gravity data, and altimetry data. The very high degree up to 2,190 of the EGM08 model, having spatial resolution of 5 arc-minutes, is based on the surface and altimetry gravity data, integrated with the satellite data of low to medium degree harmonics. However the terrestrial data sets still suffer from missing or poor quality data especially in South America^1,2^, Africa, China including the Qinghai-Tibetan plateau^3^ and its surrounding area. In these areas most of the gravity data is derived by geophysical prediction with topography data, therefore the high spatial resolution of the global combination gravity models has little meaning in such areas, including the Tibetan Plateau.

We also use sedimentary thickness data and topography data: the 30′×30′ sedimentary thickness data set^4^ provided by the Institute of Geodesy and Geophysics (IGG), Chinese Academy of Science, which is based on campaign geophysical data sources, mainly seismic data (Lithospheric Dynamic Atlas of China, 1989) and the JGP95E 5′ data that was developed to support EGM96 modeling^5^. We choose the same data sets of our previous studies^6,7^, not only because the primary purpose of this study is to investigate the improvement of the Moho and Moho fold models due to the benefit of the newly released global gravity models, but also because there has been no other improved sedimentary data nor topography data available. In addition, this study primarily focuses on the very long wavelength of Moho signals and thus the existing data sets are adequate.

We follow the analyses and methodology of our former study^7^ for upward continuation, calculating the gravity effect of topography and sediments, and various spectral analyses for Moho signal isolation and effective elastic thickness estimation. We also apply the so-called Parker-Oldenburg method^8-10^ for gravity inversion using the same previously optimized parameters^7^. However, unlike former studies, we do not have to use the upward continuation via Poisson’s integral formula^11^, because the correction for the no-terrestrial-gravity-data zone and its upward continuation are no longer necessary since EIGEN-GL04C. To compute the Moho fold models with the recent global gravity models we adopted the formerly established method^6^, which is based on the gravity inversion and the flexural isostatic analysis. The Moho folds are obtained by the residual between the gravity and the isostatic Moho and was first applied to the Tibetan plateau.

**2. The gravity anomaly and Moho topography computation**

Gravity anomaly of the study area is shown in Fig. S1. For the purpose of suppressing the high-frequency error caused by shallow layer uncertainty of terrain and sediment, the reference level is set to 7-km height above mean sea level (MSL), a little higher than maximum topography, rather than the normally used MSL. The free-air anomaly of the study area is calculated from the spherical harmonic (or Stokes’) coefficients^9^ representing the GO_CONS_GCF_2_DIR_R5 global gravity model. The range of $\Delta g$ is from –200 mGal to 232 mGal, with a mean of –7 mGal and a standard deviation of 58 mGal (Fig. S1). The edge effect, representing the combined gravity signal of topography and deeply seated crust-mantle interface, is clearly found along the boundary of the plateau while the other areas have a rather small lateral gravity variation.

The Bouguer anomaly, reflecting the subsurface density structure, is calculated from the above free-air anomaly by eliminating the gravity effect caused by the mass elements of terrain above MSL and sediments in spherical coordinates. The Bouguer anomaly varies between –559 mGal and 12 mGal, with a mean of –266 mGal and a standard deviation of 157 mGal (Fig. S1). Significantly low gravity anomaly is observed inside the plateau, having east-west directionality, representing the deep crust-mantle boundary distributed beneath the region, while the surrounding areas have higher anomaly denoting thinner crust.

The gravity differences of the EIGEN-GL04C and the GGM02C/EGM96 with the GO_CONS_GCF_2_DIR_R5 gravity model are depicted in Fig. S2, and show the improvement of the new GOCE data respect to the fields used in the previous studies^6,7^, respectively. The first difference ranges from –77 mGal to 104 mGal, with a near zero mean (–0.13 mGal), and a standard deviation of 13 mGal, while the second difference ranges from –93 mGal to 68 mGal, with a mean of –0.26 mGal and a standard deviation of 13 mGal. Large differences are located along the southern and western border of the plateau, coinciding with the area of no-terrestrial gravity data.^5,7^ The Moho topography is computed from the gravity inversion with the GO_CONS_GCF_2_DIR_R5 model. Its difference with the previous studies using earlier gravity models, EIGEN-GL04C and GGM02C/EGM96 is shown in Fig. S3. The constraints for the gravity inversion are taken to be same as those of the previous study^7^, so as to assess the improvement of GOCE-based gravity models used to invert for subsurface structure models. These parameters are density contrast, mean depth, and filtering parameters: The density parameters were based on the modified model of seismic section INDEPTH III^13^. The density of topography is assumed to be 2,570 kg/m^3^ and the sediment density to be 2,465 kg/m^3^, while the density contrast at the Moho discontinuity is set to be 368 kg/m^3^ and was optimized together with the other two values, 268 and 468 kg/m^3^ by comparing the gravity-inverted Moho undulation with various seismic experiments^14-16^. Mean depth of the Moho undulation, 47 km below MSL, was based on the widely used power spectral analysis^17^. The density parameters and reference depth are compatible with those of the previous gravity inversion^18,19^, which were selected to fit well with the constraining seismic lines. The filtering parameters for separating the Moho signals from the total Bouguer anomaly was determined from various spectral analyses and testing of several sets of parameters. So the filter passes the wavelengths longer than 310 km as Moho signals, but cuts off wavelengths shorter than 220 km, which are supposed to be upper crustal signals, while it partially passes intermediate wavelengths.

For the gravity inversion using the above parameters, we apply the so-called Parker-Oldenburg method^8-10^, which is based on the fast Fourier transform. The inversion is performed on an area (x: –1900~1910 km, y: –1260~1280 km) slightly wider than the study area (x: –1700~1700km, y: –1100~1100km) and focused on the plateau. A mirroring technique is applied to treat the Gibbs phenomenon. Convergence of the subsurface structure during iterations is monitored with the root-mean-square (RMS) difference of the Moho between successive iterations. When we set the stopping criterion to a quite small value of 1 m, we finally get the converged Moho topography model with RMS difference of 0.6 m at the 10^th^ iteration. The gravity effect of the computed Moho model showed only small discrepancies from the input gravity data with a RMS difference of 1 mGal, while slightly larger differences of –6~7 mGal are found in the narrow area of western Tibet (Fig. S4).

**3. Remarks**

We have presented the advanced three-dimensional models of the Tibetan Mohorovičić discontinuity (Moho topography and ranges) and its deformation (Moho fold) by analyzing a decade of recent global gravity models since 2004. We also showed that there was an evident improvement in disclosing the Moho features beneath the Tibetan plateau since the GOCE mission data are available.

The improvement of the Moho topography model computed by gravity inversion of GO_CONS_GCF_2_DIR_R5 is evident from the better directionality of the Moho ranges than the previous model of EIGEN-GL04C^6^, while the overall features are quite similar with the latter. The Moho fold model from the gravity model, GO_CONS_GCF_2_DIR_R5, reconfirms the improvement of the gravity model by disclosing the more evident directionality of the folds compared with the previous model computed using EIGEN-GL04C. Comparison of Moho fold models from many other global gravity models denotes that they seem to have improved year after year and converged since 2011 or 2012, with a conspicuous jump in improvement in 2010 (GO_CONS_GCF_2_TIM_R1) since the GOCE mission data have been first included. The amplitude of the Moho fold converged to the range from –9 km to 9 km with standard deviation about 2 km in the models.

Since the introduction of the GOCE data, it is observed that the N-S directional stripe patterns, due to aliasing problem of GRACE data, seem to be greatly suppressed. It is also found that the GOCE data is useful in recovering the lost Moho signals due to the too strong filter applied on the GRACE data. Finally the problem of no-terrestrial data area seems to no longer influence the study of Moho topography of the Tibetan plateau due to the higher resolution of the GOCE data: the problem was evident in earlier combination models, where the Moho was contaminated by poor quality of surface gravity data and their low accuracy. Thus it should be amended the analysis of the former study^3^ by saying, ‘… according to the resolution of GRACE gravity and power spectral densities of Bouguer anomaly and preliminary Moho models, the satellite-only data turns out to have resolution that is high enough to recover signals generated by the very deep Moho beneath Tibet’. It is because that they seem to have inevitable problem either they are losing Moho signals by smoothing in GRACE satellite-only models or they are affected by the wide area of no or bad quality data in combination models, even though they are advertising to have higher harmonics signals.

Thus the three-dimensional Tibetan Moho topography, ranges, and fold model of this study from the most recent GOCE mission-based global gravity data could be, at present, considered the state-of-the-art solution for the Tibetan crust and should influence the study of other collisional boundaries as well.

**Supplementary References**

1. Alvarez O., Gimenez M., Braitenberg C. & Folguera A. GOCE satellite derived gravity and gavity gradient corrected for topographic effect in the South Central Andes Region. *Geophys. J. Int.* **190**, 941-959; DOI:10.1111/j.1365-246X.2012.05556.x (2012).

2. Bomfim E. P., Braitenberg C. & Molina E. C. Mutual evaluation of global gravity models (EGM2008 and GOCE) and terrestrial data in Amazon Basin, Brazil. *Geophys. J. Int.* **195**, 870-882; DOI:10.1093/gji/ggt283 (2013).

3. Fecher, T., Pail, R. & Gruber, T. Global gravity field modeling based on GOCE and complementary gravity data. *Int. J. Appl. Earth Obs. Geoinfo.* **35**, 120-127; DOI:10.1016/j.jag.2013.10.005 (2015).

*4. Lithospheric Dynamic Atlas of China*. China Cartographic Publishing House, Beijing (1989).

5. Lemoine, F. G. et al. *The Development of the Joint NASA GSFC and the Mational Imagery and mapping Agency (NIMA) Geopotential Model EGM96*, NASA/TP-1998-206861 (1998).

6. Shin, Y. H. et al. Three-dimensional fold structure of the Tibetan Moho from GRACE gravity data. *Geophys. Res. Lett.* **36**, L01302; DOI:10.1029/2008GL036068 (2009).

7. Shin, Y. H., Xu, H., Braitenberg, C., Fang, J. & Wang, Y. Moho undulations beneath Tibet from GRACE-integrated gravity data. *Geophys. J. Int.* **170**, 971-985; DOI: 10.1111/j.1365-246X.2007.03457.x (2007).

8. Parker, R. L. The Rapid Calculation of Potential Anomalies. *Geophys. J. R. Astr. Soc*. **31**, 447-455; DOI: 10.1111/j.1365-246X.1973.tb06513.x (1972).

9. Oldenburg, D. W. The Inversion and Interpretation of Gravity Anomalies. *Geophysics* **39**, 526-536; DOI: 10.1190/1.1440444 (1974).

10. Shin, Y. H., Choi, K. S. & Xu, H. Three-dimensional Forward and Inverse Models for Gravity Fields Based on the Fast Fourier Transform. *Com. & Geosci*. **32**, 727-738; DOI: 10.1016/j.cageo.2005.10.002 (2006).

11. Heiskanen, W. A. & Moritz, H. *Physical Geodesy*. Institute of Physical Geodesy, Technical University, Graz, Austria (1987).

12. Cruz, J. Y. & Laskowski, P. *Upward continuation of surface gravity anomalies*. Dept. of Geodetic Science and Surveying, Ohio State Univ., Report No. 360 (1984).

13. Haines, S. S. et al. INDEPTH III seismic data: From surface observations to deep crustal processes in Tibet*. Tectonics* **22**, 1001; DOI:10.1029/2001TC001305 (2003).

14. Kind, R. et al. Seismic Images of Crust and Upper Mantle Beneath Tibet: Evidence for Eurasian Plate Subduction. *Science* **298**, 1219-1221; DOI: 10.1126/science.1078115 (2002).

15. Mitra, S., Priestley, K., Bhattacharyya, A. & Gaur, V. K. Crutsal structure and earthquake focal depths beneath northeastern India and southern Tibet. *Geophys. J. Int.* **160**, 227-248; DOI: 10.1111/j.1365-246X.2004.02470.x (2005).

16. Zhao, J. et al. Crustal structure across the Altyn Tagh Range at the northern margin of the Tibetan plateau and tectonic implications. *Earth Planet. Sci. Lett*. **241**, 804-814; DOI: 10.1016/j.epsl.2005.11.003 (2006).

17. Spector, A., & Grant, F. S. Statistical models for interpreting aeromagnetic data. *Geophysics* **35**, 293-302; DOI: 10.1190/1.1440092 (1970).

18. Braitenberg, C., Zadro, M., Fang, J., Wang, Y. & Hsu, H. T. Gravity Inversion in Qinghai-Tibet Plateau. *Phys. Chem. Earth (A)* **25**, 381-386; DOI: 10.1016/S1464-1895(00)00060-0 (2000).

19. Braitenberg, C., Zadro, M., Fang, J., Wang, Y. & Hsu, H. T. The gravity and isostatic Moho undulations in Qinghai-Tibet Plateau. *J. Geodyn.* **30**, 489-505; DOI: 10.1016/S0264-3707(00)00004-1 (2000).

**Supplementary Figures**


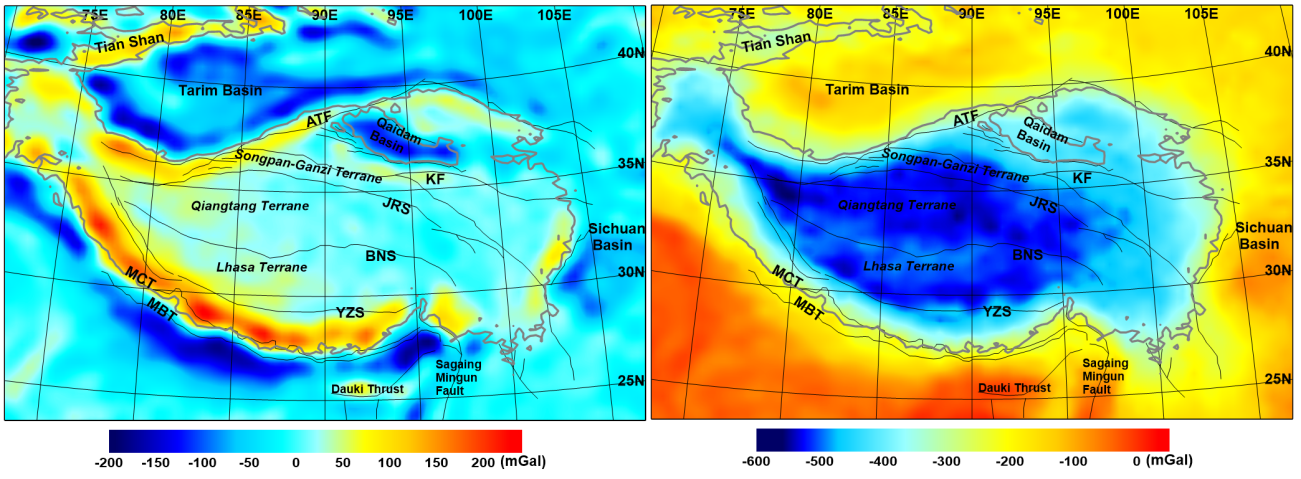


Figure S1. Free-air anomaly (left) and Bouguer anomaly (right) of GO_CONS_GCF_2_DIR_R5 at 7-km height above mean sea level. (The figure is generated using Surfer (http://goldensoftware.com/) by Young Hong Shin)


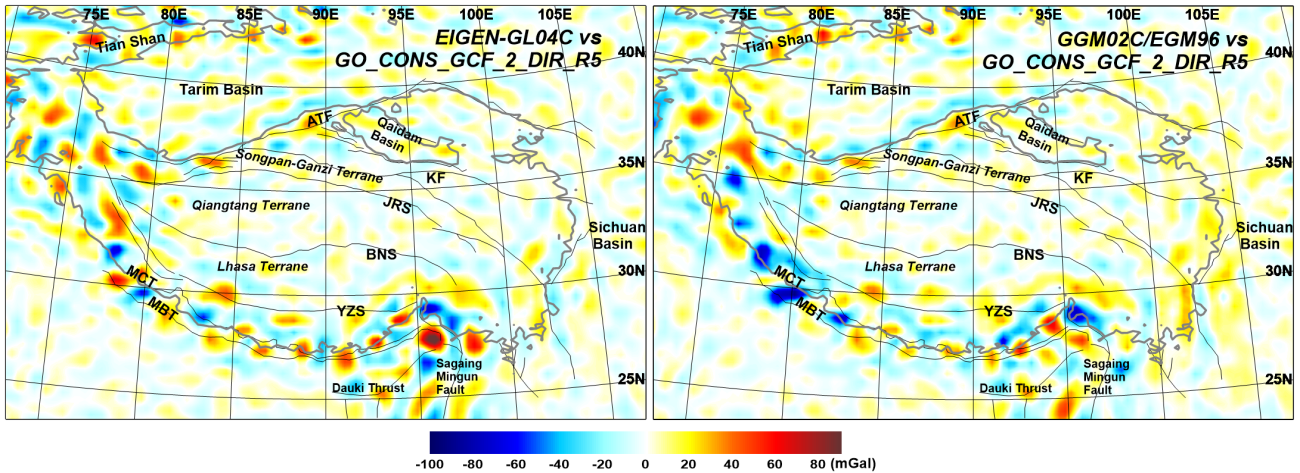


Figure S2. Difference of gravity derived from EIGEN-GL04C (left) and GGM02C/EGM96 (right), with respect to the one from GO_CONS_GCF_2_DIR_R5 models. (The figure is generated using Surfer (http://goldensoftware.com/) by Young Hong Shin)


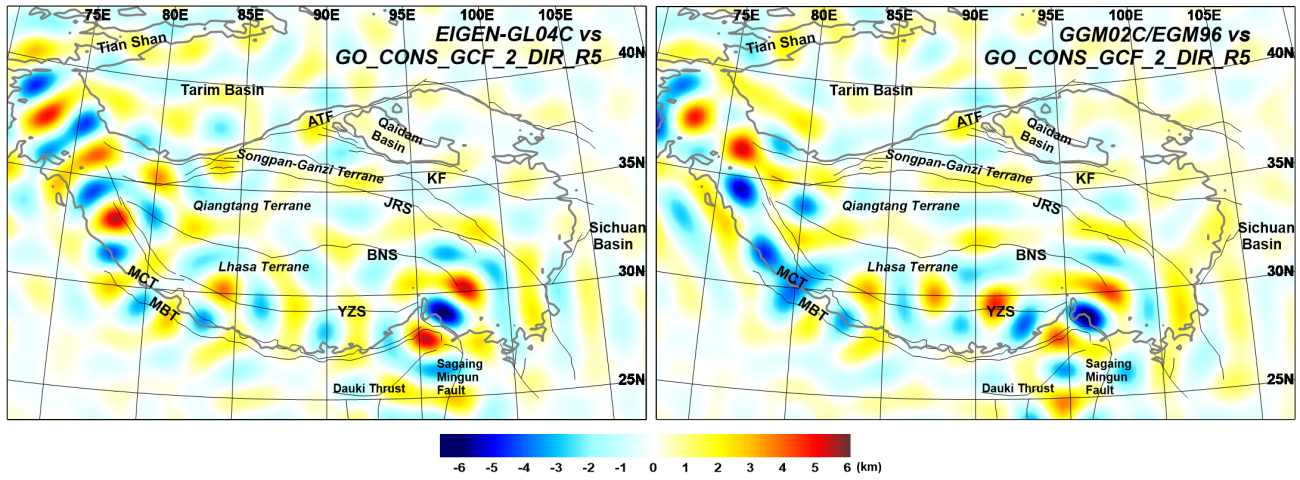


Figure S3. Difference of Moho topography derived from EIGEN-GL04C (left) and GGM02C/EGM96 (right), with respect to the one from GO_CONS_GCF_2_DIR_R5 models. (The figure is generated using Surfer (http://goldensoftware.com/) by Young Hong Shin)


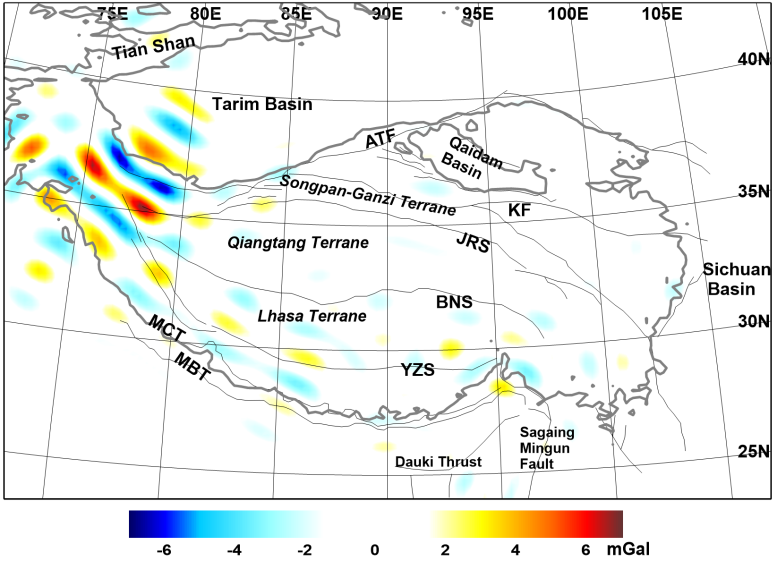


Figure S4. Gravity residual of the gravity inversion. (The figure is generated using Surfer (http://goldensoftware.com/) by Young Hong Shin)


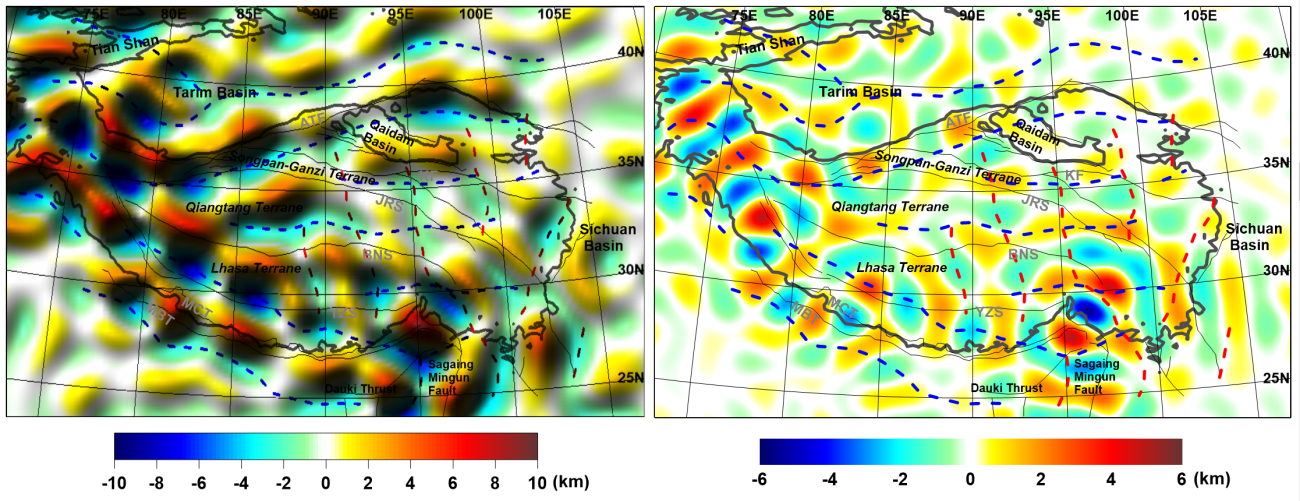


Figure S5. A shaded relief map of the Moho Folds derived from EIGEN-GL04C gravity model (left) and its difference with that from GO_CONS_GCF_2_DIR_R5 gravity model (right). EW and NS directionality of Moho fold troughs (blue and red dashed-lines) show slight misfit with the structure, especially in eastern Tibet, because the dashed-lines are depicted to fit better to those from GO_CONS_GCF_2_DIR_R5. (The figure is generated using Surfer (http://goldensoftware.com/) by Young Hong Shin)

**Supplementary Tables**

Table S1 Recent global gravity models.

| Year | Satellite-only Model | | | | | | | | | | Combination Model w/  Surface & Altimetry | | | |
| --- | --- | --- | --- | --- | --- | --- | --- | --- | --- | --- | --- | --- | --- | --- |
|  | GRACE etc. (w/o GOCE) | | | GOCE, GRACE, LAGEOS, etc. | | | | GOCE-only | | |  |  |  |  |
|  | Gravity  Model | Nmax | GRACE  (year) | Gravity  Model | Nmax | GOCE  (month) | GRACE  (year) | Gravity  Model | Nmax | GOCE  (month) | Gravity  Model | Nmax | GOCE  (month) | GRACE  (year) |
| 2014 |  |  |  | DIR-R5 | 300 | 48 | 10 | TIM-R5 | 280 | 48 |  |  |  |  |
|  | GGM05S | 180 | 10 | *EIGEN-6S2* | *260* | 35 | 9.7 |  |  |  | EIGEN-6C3stat | 1949 | 35 | 9.7 |
| 2013 | *Tongji-GRACE01* | *160* | *4.1* |  |  |  |  | *ITG-GOCE02* | *240* | 8 |  |  |  |  |
|  |  |  |  | DIR-R4 | 260 | 34 | 10 | TIM-R4 | 250 | 32 |  |  |  |  |
| 2012 |  |  |  | *DGM-1S* | *250* | *14* | *7* |  |  |  | EIGEN-6C2 | 1949 | 11.5 | 7.8 |
|  |  |  |  | GOCO03S | 250 | 18 | 7 |  |  |  |  |  |  |  |
| 2011 |  |  |  | DIR-R3 | 240 | 17.6 | 6.3 | TIM-R3 | 250 | 17.6 | EIGEN-6C | 1420 | 6.7 | 6.5 |
|  |  |  |  | EIGEN-6S | 240 | 6.7 | 6.5 |  |  |  | GIF48 | 360 |  | 5.5 |
|  | *AIUB-GRACE03* | *160* | *6.1* | GOCO02S | 250 | 8 | 7 |  |  |  |  |  |  |  |
|  |  |  |  | DIR-R2 | 240 | 8 | ITG-GRACE2010S | TIM-R2 | 250 | 8 |  |  |  |  |
| 2010 |  |  |  | DIR-R1 | 240 | 2.4 |  | TIM-R1 | 224 | 2.4 |  |  |  |  |
|  | ITG-GRACE2010S | 180 | 7 | GOCO01S | 224 | 2 | 7 |  |  |  | EIGEN-51C | 359 |  | 6 |
| 2009 | *AIUB-GRACE02* | *150* |  |  |  |  |  |  |  |  | GGM03C | 360 |  | 3.9 |
| 2008 | GGM03S | 180 | 3.9 |  |  |  |  |  |  |  |  |  |  |  |
|  | *AIUB-GRACE01* | *120* |  |  |  |  |  |  |  |  |  |  |  |  |
|  | EIGEN-5S | 150 | 4.5 |  |  |  |  |  |  |  | EIGEN-5C | 360 |  | 4.5 |
|  |  |  |  |  |  |  |  |  |  |  | EGM2008 | 2190 |  | ITG-GRACE03S |
| 2007 | ITG-GRACE03S | 180 | 4.7 |  |  |  |  |  |  |  |  |  |  |  |
| 2006 | *ITG-GRACE02S* | *170* |  |  |  |  |  |  |  |  |  |  |  |  |
|  | EIGEN-GL04S1 | 150 | 2.3 |  |  |  |  |  |  |  | EIGEN-GL04C | 360 |  | 2.3 |
| 2005 |  |  |  |  |  |  |  |  |  |  | *EIGEN-CG03C* | *360* |  |  |
| 2004 | *GGM02S* | *160* | *1* |  |  |  |  |  |  |  | GGM02C | 200 |  | 1 |
|  | *EIGEN-GRACE02S* | *150* |  |  |  |  |  |  |  |  | *EIGEN-CG01C* | *360* |  |  |
| *TIM and DIR represent the GO_CONS_GCF_2_TIM and GO_CONS_GCF_2_DIR models, respectively.* | | | | | | | | | | | | | | |

Table S2. Statistics of Moho topography models

| Year | Satellite-only Model | | | | | | | | | | | | Combination Model w/  Surface & Altimetry | | | |
| --- | --- | --- | --- | --- | --- | --- | --- | --- | --- | --- | --- | --- | --- | --- | --- | --- |
|  | GRACE etc. (w/o GOCE) | | | | GOCE, GRACE, LAGEOS, etc. | | | | GOCE-only | | | |  |  |  |  |
|  | Gravity  Model | STD  (km) | Max  (km) | Min  (km) | Gravity  Model | STD  (km) | Max  (km) | Min  (km) | Gravity  Model | STD  (km) | Max  (km) | Min  (km) | Gravity  Model | STD  (km) | Max  (km) | Min  (km) |
| 2014 | GGM05S | 12.24 | -29.18 | -81.61 | DIR-R5 | 12.23 | -29.10 | -81.55 | TIM-R5 | 12.23 | -29.12 | -81.57 | EIGEN-6C3stat | 12.23 | -29.08 | -81.48 |
| 2013 |  |  |  |  | DIR-R4 | 12.23 | -29.09 | -81.60 | TIM-R4 | 12.23 | -29.13 | -81.60 |  |  |  |  |
| 2012 |  |  |  |  | GOCO03S | 12.23 | -29.15 | -81.60 |  |  |  |  | EIGEN-6C2 | 12.23 | -29.07 | -81.44 |
| 2011 |  |  |  |  | DIR-R3 | 12.23 | -29.08 | -81.62 | TIM-R3 | 12.23 | -29.14 | -81.53 |  |  |  |  |
|  |  |  |  |  | EIGEN-6S | 12.23 | -29.05 | -81.57 |  |  |  |  | EIGEN-6C | 12.23 | -29.09 | -81.25 |
|  |  |  |  |  | GOCO02S | 12.23 | -29.18 | -81.59 |  |  |  |  | GIF48 | 12.23 | -28.78 | -80.45 |
|  |  |  |  |  | DIR-R2 | 12.23 | -29.17 | -81.54 | TIM-R2 | 12.23 | -29.19 | -81.50 |  |  |  |  |
| 2010 |  |  |  |  | DIR-R1 | 11.76 | -29.95 | -76.47 | TIM-R1 | 12.23 | -29.24 | -81.57 |  |  |  |  |
|  | ITG-GRACE2010S | 11.76 | -30.03 | -76.75 | GOCO01S | 11.76 | -29.98 | -76.77 |  |  |  |  | EIGEN-51C | 11.76 | -29.57 | -75.26 |
| 2009 |  |  |  |  |  |  |  |  |  |  |  |  | GGM03C | 12.23 | -29.22 | -81.19 |
| 2008 | GGM03S | 12.28 | -29.22 | -80.65 |  |  |  |  |  |  |  |  | EIGEN-5C | 12.22 | -27.81 | -81.54 |
|  | EIGEN-5S | 12.25 | -28.64 | -80.99 |  |  |  |  |  |  |  |  | EGM2008 | 12.20 | -28.93 | -78.74 |
| 2007 | ITG-GRACE03S | 12.23 | -29.28 | -81.91 |  |  |  |  |  |  |  |  |  |  |  |  |
| 2006 | EIGEN-GL04S1 | 12.27 | -28.06 | -81.77 |  |  |  |  |  |  |  |  | EIGEN-GL04C | 12.25 | -28.11 | -83.23 |
| 2004 |  |  |  |  |  |  |  |  |  |  |  |  | GGM02C | 12.20 | -29.00 | -79.47 |
| *Average of the Moho topography is -49.6 km* | | | | | | | | | | | | | | | | |

Table S3. Difference of Moho topography models with the model obtained from the GO_CONS_GCF_2_DIR_R5 gravity model

| Year | Satellite-only Model | | | | | | | | | | | | Combination Model w/  Surface & Altimetry | | | |
| --- | --- | --- | --- | --- | --- | --- | --- | --- | --- | --- | --- | --- | --- | --- | --- | --- |
|  | GRACE etc. (w/o GOCE) | | | | GOCE, GRACE, LAGEOS, etc. | | | | GOCE-only | | | |  |  |  |  |
|  | Gravity  Model | STD  (km) | Max  (km) | Min  (km) | Gravity  Model | STD  (km) | Max  (km) | Min  (km) | Gravity  Model | STD  (km) | Max  (km) | Min  (km) | Gravity  Model | STD  (km) | Max  (km) | Min  (km) |
| 2014 | GGM05S | 0.41 | 1.47 | -1.74 | DIR-R5 | - | - | - | TIM-R5 | 0.02 | 0.07 | -0.06 | EIGEN-6C3stat | 0.07 | 0.22 | -0.22 |
| 2013 |  |  |  |  | DIR-R4 | 0.03 | 0.24 | -0.19 | TIM-R4 | 0.03 | 0.30 | -0.18 |  |  |  |  |
| 2012 |  |  |  |  | GOCO03S | 0.06 | 0.54 | -0.44 |  |  |  |  | EIGEN-6C2 | 0.11 | 0.33 | -0.36 |
| 2011 |  |  |  |  | DIR-R3 | 0.06 | 0.42 | -0.32 | TIM-R3 | 0.04 | 0.36 | -0.27 |  |  |  |  |
|  |  |  |  |  | EIGEN-6S | 0.19 | 1.32 | -1.32 |  |  |  |  | EIGEN-6C | 0.21 | 1.61 | -1.37 |
|  |  |  |  |  | GOCO02S | 0.06 | 0.55 | -0.42 |  |  |  |  | GIF48 | 0.45 | 2.07 | -2.72 |
|  |  |  |  |  | DIR-R2 | 0.18 | 1.32 | -1.58 | TIM-R2 | 0.06 | 0.37 | -0.27 |  |  |  |  |
| 2010 |  |  |  |  | DIR-R1 | 1.02 | 5.51 | -3.84 | TIM-R1 | 0.10 | 0.66 | -0.56 |  |  |  |  |
|  | ITG-GRACE2010S | 1.00 | 5.20 | -3.60 | GOCO01S | 0.99 | 5.19 | -3.59 |  |  |  |  | EIGEN-51C | 1.16 | 6.48 | -5.24 |
| 2009 |  |  |  |  |  |  |  |  |  |  |  |  | GGM03C | 0.82 | 3.94 | -4.45 |
| 2008 | GGM03S | 1.08 | 3.52 | -3.51 |  |  |  |  |  |  |  |  | EIGEN-5C | 1.27 | 6.28 | -6.67 |
|  | EIGEN-5S | 1.02 | 3.71 | -3.78 |  |  |  |  |  |  |  |  | EGM2008 | 0.96 | 5.38 | -7.45 |
| 2007 | ITG-GRACE03S | 0.37 | 1.66 | -1.47 |  |  |  |  |  |  |  |  |  |  |  |  |
| 2006 | EIGEN-GL04S1 | 1.16 | 4.44 | -4.54 |  |  |  |  |  |  |  |  | EIGEN-GL04C | 1.12 | 5.70 | -6.22 |
| 2004 |  |  |  |  |  |  |  |  |  |  |  |  | GGM02C | 1.15 | 5.43 | -6.56 |

Table S4. Statistics of estimated Moho fold models.

| Year | Satellite-only Model | | | | | | | | | | | | Combination Model w/  Surface & Altimetry | | | |
| --- | --- | --- | --- | --- | --- | --- | --- | --- | --- | --- | --- | --- | --- | --- | --- | --- |
|  | GRACE etc. (w/o GOCE) | | | | GOCE, GRACE, LAGEOS, etc. | | | | GOCE-only | | | |  |  |  |  |
|  | Gravity  Model | STD  (km) | Max  (km) | Min  (km) | Gravity  Model | STD  (km) | Max  (km) | Min  (km) | Gravity  Model | STD  (km) | Max  (km) | Min  (km) | Gravity  Model | STD  (km) | Max  (km) | Min  (km) |
| 2014 | GGM05S | 1.93 | 8.92 | -8.91 | DIR-R5 | 1.89 | 8.82 | -8.87 | TIM-R5 | 1.89 | 8.85 | -8.90 | EIGEN-6C3stat | 1.89 | 8.95 | -8.89 |
| 2013 |  |  |  |  | DIR-R4 | 1.89 | 8.85 | -8.91 | TIM-R4 | 1.89 | 8.85 | -8.91 |  |  |  |  |
| 2012 |  |  |  |  | GOCO03S | 1.90 | 8.84 | -8.93 |  |  |  |  | EIGEN-6C2 | 1.89 | 9.00 | -8.87 |
| 2011 |  |  |  |  | DIR-R3 | 1.89 | 8.90 | -8.95 | TIM-R3 | 1.89 | 8.84 | -8.86 |  |  |  |  |
|  |  |  |  |  | EIGEN-6S | 1.88 | 9.00 | -8.99 |  |  |  |  | EIGEN-6C | 1.88 | 9.06 | -8.73 |
|  |  |  |  |  | GOCO02S | 1.90 | 8.85 | -8.93 |  |  |  |  | GIF48 | 1.89 | 9.15 | -8.42 |
|  |  |  |  |  | DIR-R2 | 1.88 | 8.90 | -8.90 | TIM-R2 | 1.89 | 8.86 | -8.83 |  |  |  |  |
| 2010 |  |  |  |  | DIR-R1 | 1.26 | 5.93 | -5.54 | TIM-R1 | 1.90 | 8.94 | -8.88 |  |  |  |  |
|  | ITG-GRACE2010S | 1.28 | 5.91 | -5.81 | GOCO01S | 1.27 | 5.97 | -5.82 |  |  |  |  | EIGEN-51C | 1.24 | 6.12 | -5.23 |
| 2009 |  |  |  |  |  |  |  |  |  |  |  |  | GGM03C | 1.94 | 8.67 | -8.56 |
| 2008 | GGM03S | 2.12 | 8.46 | -7.72 |  |  |  |  |  |  |  |  | EIGEN-5C | 1.92 | 9.52 | -8.94 |
|  | EIGEN-5S | 2.00 | 8.69 | -8.97 |  |  |  |  |  |  |  |  | EGM2008 | 1.81 | 9.08 | -8.88 |
| 2007 | ITG-GRACE03S | 1.93 | 8.65 | -9.03 |  |  |  |  |  |  |  |  |  |  |  |  |
| 2006 | EIGEN-GL04S1 | 2.09 | 8.96 | -9.62 |  |  |  |  |  |  |  |  | EIGEN-GL04C | 2.04 | 9.02 | -10.14 |

Table S5. Difference of Moho fold models between other gravity field models and the GO_CONS_GCF_2_DIR_R5 reference model

| Year | Satellite-only Model | | | | | | | | | | | | Combination Model w/  Surface & Altimetry | | | |
| --- | --- | --- | --- | --- | --- | --- | --- | --- | --- | --- | --- | --- | --- | --- | --- | --- |
|  | GRACE etc. (w/o GOCE) | | | | GOCE, GRACE, LAGEOS, etc. | | | | GOCE-only | | | |  |  |  |  |
|  | Gravity  Model | STD  (km) | Max  (km) | Min  (km) | Gravity  Model | STD  (km) | Max  (km) | Min  (km) | Gravity  Model | STD  (km) | Max  (km) | Min  (km) | Gravity  Model | STD  (km) | Max  (km) | Min  (km) |
| 2014 | GGM05S | 0.38 | 1.38 | -1.66 | DIR-R5 | - | - | - | TIM-R5 | 0.02 | 0.07 | -0.06 | EIGEN-6C3stat | 0.07 | 0.20 | -0.19 |
| 2013 |  |  |  |  | DIR-R4 | 0.02 | 0.23 | -0.18 | TIM-R4 | 0.03 | 0.29 | -0.17 |  |  |  |  |
| 2012 |  |  |  |  | GOCO03S | 0.05 | 0.52 | -0.44 |  |  |  |  | EIGEN-6C2 | 0.10 | 0.29 | -0.33 |
| 2011 |  |  |  |  | DIR-R3 | 0.05 | 0.41 | -0.31 | TIM-R3 | 0.04 | 0.34 | -0.25 |  |  |  |  |
|  |  |  |  |  | EIGEN-6S | 0.17 | 1.19 | -1.18 |  |  |  |  | EIGEN-6C | 0.19 | 1.45 | -1.23 |
|  |  |  |  |  | GOCO02S | 0.06 | 0.53 | -0.42 |  |  |  |  | GIF48 | 0.40 | 1.79 | -2.46 |
|  |  |  |  |  | DIR-R2 | 0.16 | 1.18 | -1.40 | TIM-R2 | 0.05 | 0.35 | -0.26 |  |  |  |  |
| 2010 |  |  |  |  | DIR-R1 | 0.67 | 3.33 | -3.16 | TIM-R1 | 0.09 | 0.62 | -0.55 |  |  |  |  |
|  | ITG-GRACE2010S | 0.65 | 3.10 | -3.00 | GOCO01S | 0.65 | 3.05 | -2.91 |  |  |  |  | EIGEN-51C | 0.82 | 4.13 | -4.12 |
| 2009 |  |  |  |  |  |  |  |  |  |  |  |  | GGM03C | 0.73 | 3.56 | -3.98 |
| 2008 | GGM03S | 1.00 | 3.24 | -3.26 |  |  |  |  |  |  |  |  | EIGEN-5C | 1.11 | 5.54 | -5.75 |
|  | EIGEN-5S | 0.94 | 3.46 | -3.47 |  |  |  |  |  |  |  |  | EGM2008 | 0.79 | 4.23 | -6.04 |
| 2007 | ITG-GRACE03S | 0.34 | 1.50 | -1.46 |  |  |  |  |  |  |  |  |  |  |  |  |
| 2006 | EIGEN-GL04S1 | 1.07 | 4.05 | -4.15 |  |  |  |  |  |  |  |  | EIGEN-GL04C | 0.97 | 4.99 | -5.40 |
